# The impact of the COVID-19 pandemic on cancer care in the public health subsector, province of Santa Fe, Argentina

**DOI:** 10.3332/ecancer.2021.1270

**Published:** 2021-07-26

**Authors:** Graciela Lopez de Degani, Leandro Duarte, Julia Ismael, Laura Martinez, Florencia López

**Affiliations:** 1 Cancer Control Agency, Ministry of Health, Bv. Pellegrini 3551, Zip Code 3000, Santa Fe, Argentina

**Keywords:** COVID-19, pandemic, cancer, Santa Fe, public health

## Abstract

**Introduction:**

The severe acute respiratory syndrome coronavirus 2 pandemic coronavirus disease (COVID-19) and the measures taken to lessen its impact have had side effects affecting timely care of other diseases. The aim of this paper is to quantify the impact of the pandemic on the cancer care line in the province of Santa Fe, Argentina.

**Method:**

It is an observational cross-sectional study comparing the impact on selected variables of the pre-pandemic and intra-pandemic periods. The formula of percentage variation was used to show the differences. The positivity index was calculated and expressed as a percentage. The proportions of both periods were compared through the chi-squared test and its *p*-value.

**Results:**

Reductions were observed in all the variables under study. However, the deeper impact was evident in screening, with 56%–87% decreases in the number of procedures carried out. A 26% reduction was seen in diagnosis. Treatment was the variable with the least impact, with a 3% decrease.

**Discussion:**

COVID-19 as well as the measures taken to reduce its impact caused alterations in the cancer care line in the province, with clear differences according to the variable under study. Measures related to cancer screening were displaced, prioritising the care of patients already diagnosed and treated.

**Conclusion:**

Considering the new increase in the number of COVID-19 cases, it is essential to adapt the healthcare system, and design new innovative strategies to reduce long-term consequences.

## Background

Both the pandemic caused by the new coronavirus severe acute respiratory syndrome coronavirus 2 coronavirus disease (COVID-19) and the measures taken to lessen its impact have undoubtedly had an effect on the management of other diseases, especially chronic non-communicable diseases [1–4].

Cancer is a public health issue in Latin America. Listed under non-communicable chronic diseases, it has been positioned as the second leading cause of death in Argentina [5], after cardiovascular diseases, which implies a high demand for care and healthcare resources.

Several studies suggest that cancer patients are more likely to develop COVID-19-related complications and a more severe form of the disease [6–8], as well as experience reduced or delayed cancer care [9, 10]. A greater impact in low-income countries and some worsening of existing difficulties and consequences in the medium and long term are expected [10]. Some authors call it the third wave [12].

This impact is depicted in several international publications, with a reduced percentage in the number of screening studies carried out, ranging from 36% to 96%, although the local data available are scarce [13].

The province of Santa Fe in the Argentine Republic is the third-most densely populated in the country. Under the Ministry of Health, the Cancer Control Agency (CCA) is the management authority for cancer control for the entire territory of the province. The CCA has 12 programmes aimed at reducing the mortality associated with this condition, mainly based on the line of care including screening, timely and qualitative early diagnosis and treatment and palliative care. Our country has designed, planned and launched a comprehensive and systematic National Cancer Control Plan [NCCP], which is in force (2018–2022) [14]. The objectives on population screening programme coverage (according to the World Health Organization guidelines) suggested by the NCCP constituted a formidable challenge for the province, even before the pandemic.

Three national screening programmes for breast, cervical and colorectal cancer have been implemented in the province. The pre-pandemic coverage percentages of breast cancer screening with mammograms by province does not exceed 50% in the best of cases (provincial average = 18%); cervical screening with Papanicolaou (PAP) does not reach 40% (provincial average = 27%); and the immunochemical faecal occult blood test (iFOBT) for colon cancer screening does not reach sufficient figures to modify the mortality statistics (provincial average = 2%). This problem is not exclusive to this jurisdiction, and is seen throughout the country and in Latin America as well [15, 16].

Since the COVID-19 pandemic broke out, the healthcare system has focused on this emerging condition. As effectors and healthcare providers are decimated due to high infection rates, healthcare systems operate at a limited capacity and the first level of healthcare has collapsed; cancer and other chronic diseases have ceased to be a priority in the healthcare agenda and in the general population.

In March 2020, when the virus started circulating in the country, the National Government issued Decree 297/2020 stating a lockdown, an exceptional measure in a critical context to protect public health against the spread of the new coronavirus which lasted until Monday, November 9. This measure implied that all the people who lived, or temporarily living, in the jurisdictions where this regulation was applicable had to stay at home, and were allowed to go out only to stock up on cleaning supplies, medication and food. This public policy reduced the impact of COVID-19 in the province but brought about other complications.

The impact of the pandemic is expected to affect the entire cancer care line, from screening to treatment. Therefore, it is necessary to evaluate several points along this line to provide a more complete representation of this phenomenon.

The aim of this report is to give a global vision of the impact of the COVID-19 pandemic as well as the measures taken to protect public healthcare, in the cancer care line of the public healthcare subsector of the province of Santa Fe., Argentina.

## Methods

An observational, cross-sectional study was conducted in two periods. The analysis time was determined from the start of the lockdown (19/03/2020) to 6 months until September 2020. For the purposes of this paper, it is called **Period 2,** and was compared with the same period of the previous pre-pandemic year (19/03/2019–19/09/2019), which is called **Period 1**.

Three components from the continuous cancer care line were selected: screening section, cancer diagnosis section and specific treatment section.

Nine variables were selected to represent the results. For the screening section, the selected variables were total number of PAPs carried out and the total number of PAP smears with unsatisfactory results (including Atypical Squamous Cells of Undetermined Significance [ASCUS], high- and low-grade dysplasia or invasive cancer); number of mammograms carried out and total number of positive results (Breast Imaging Reporting and Data System [BI-RADS]-IV or more); and the total number of iFOBTs carried out and those with positive results. A positivity index expressed as a percentage was calculated to compare between periods, with a confidence interval (CI) of 95%.

A primary database ‘SITAM’, the National Screening Information System (of its acronym in Spanish: ‘*Sistema de Información de Tamizaje*’) was used. The breast, colon and cervical cancer modules were used. A total of 25% of the provincial public mammograph providers did not report to the registry, so these data were excluded from the analysis in both periods.

For the diagnosis section of the line of care, the total number of biopsies and total number of biopsies with a positive result for cancer were selected. The source was the Santa Fe Cancer Registry (RECASFE), selected public effectors from the entire province and the total number of positive biopsies in both periods. 100% of the public pathological anatomy centres in the province report data to RECASFE (ten recording units). Two units were excluded because of the unavailability of comparative data for both periods. The positivity index was calculated and expressed as percentage.

For the specific treatment section, the total number of oncological prescriptions was used to account for the treatments carried out in patients with a diagnosis. The data source used was the Oncological Drug Prescription System (Prescripción de Medicamentos Oncológicos) of the province of Santa Fe, selecting all the prescribed treatments of cytotoxic chemotherapy, immunotherapy and target treatments for oncological diseases in the periods analysed.

To show the differences between both periods, the formulas of absolute variation (VA) VA = (Np2−Np1) and percentage variation (VP) VP = [(VA)/Np1] × 100 were used. VA is the difference between the number of screenings for the pandemic period (Np2), and the number of screenings for the previous period (Np1). Dividing this difference by Np1 and multiplying by 100 the VP expressed in % was obtained. This shows the relative percentage change compared to the first period. Negative percentages are interpreted as a reduction percentage in relation to the first period. CIs (95%) were calculated for each proportion with the Epidat 3.0 epidemiological package.

The prevalence of positivity of screening and diagnostic tests was also calculated, and the proportions of both periods were compared through the chi-squared test and its *p*-value.

Graphs and calculations were made using Microsoft Excel.

Submittal before the ethics committee was not required because personal data or the participation of any person in this study was not included.

## Results

The impact of the COVID-19 pandemic and the measures to avoid it have negatively modified the line of cancer care in the province, with reductions in all the analysed variables (Table 1).

In relation to the breast, colon and cervical cancer screening programmes, reductions were seen in the total number of procedures carried out. The total numbers of PAPs in both periods decreased by 56% (28,424 fewer PAPs). If only the unsatisfactory PAP results are considered, i.e. including those with ASCUS, high- and low-grade dysplasia or invasive cancer, in absolute numbers they were 265 PAPs less. Globally, PAP positivity index experienced a reduction of 83.79%, with a statistically significant difference (*p* = <0.0001).

The total number of mammographs decreased by 78.85% with 7,820 fewer studies between both periods. The positivity index between both periods yielded a positive result of 301.83% (*p* = <0.0001).

Regarding colorectal cancer screening, there was a significant reduction in the number of iFOBT, with a reduction of 87.80% between periods. The percentage of positivity in this case showed a reduction of 25.49%, although the value was not statistically significant (*p* = 0.3097).

In the diagnostic variable analysis, there was a reduction in the total number of biopsies carried out throughout the province, with a 40.17% reduction as compared to the previous period.

The number of positive biopsies for cancer was also reduced. The positivity index was 22.72% (*p* = <0.0001). This will be discussed in the discussion section.

If we analysed patients with a diagnosis and who are undergoing specific cancer treatments, which involves the use of cytotoxic chemotherapy, immunotherapy and target treatments, the reduction between both periods is 3.16%, with 96 fewer prescriptions. A total of 3,036 patients in treatment in period 1 and 2,940 patients in period 2 were included.

## Discussion

As of March 2021, 230,000 cases of COVID-19 and 4,158 COVID-19-related deaths were documented in the province of Santa Fe, Agentina [17].

The introduction of the mandatory lockdown was a necessary decision to contain the rapid spread of the new virus, which led to a flattening of the contagion curve [18]. In the province of Santa Fe, both the teams and the care networks were reorganised. COVID and non-COVID effectors were defined to reduce contact between infected people and vulnerable patients and avoid complications. Similarly, in the south of the province, paediatric cancer patients were grouped into a single effector.

However, the application of lockdown seen as medical technology entails collateral adverse events, which is poorly quantified so far.

Also, events stemming from the adaptation of the healthcare system and from COVID-19 itself on chronic non-communicable diseases were identified.

As mentioned above, screening, imaging, laboratory tests, pathology, surgery, systemic therapy and palliative care are essential components in cancer management. While building a line of care with these components, we have identified points from which we obtained comparative data in the province to evaluate the impact of a global event, such as the COVID-19 pandemic: screening, pathological diagnosis and systemic treatment. Screening tests as the main variable of screening; biopsies as the most important variable to define the status of cancer diagnosis; and systemic treatment as the most important variable and feasible tool to measure in the care of cancer patients.

**Figure 1.**
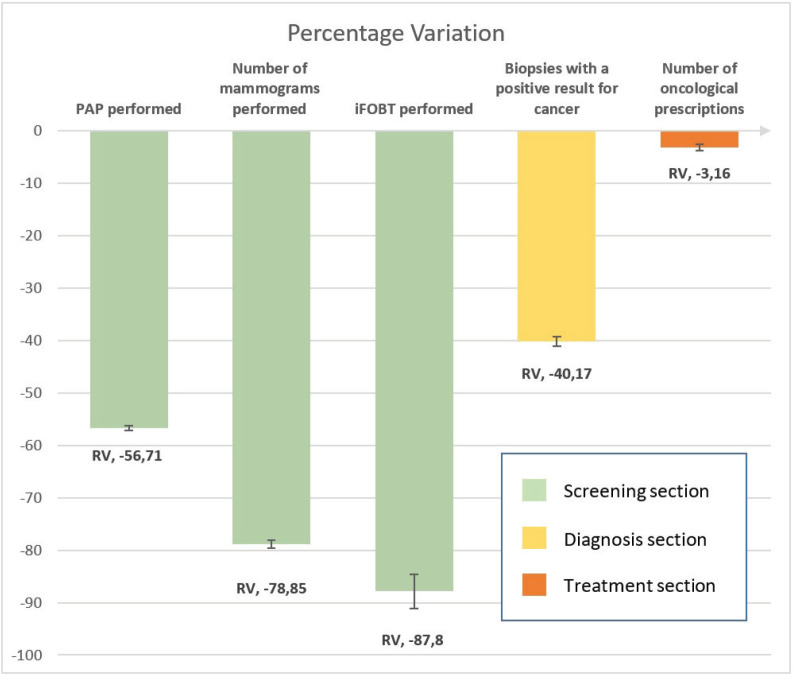
Impact of the COVID-19 Pandemic on the cancer care line. Source: Own elaboration based on data collected.

A negative impact of the COVID-19 pandemic and associated measures was observed in the cancer care line. This impact is different in different sections, mainly in the screening section compared to the other two (see Figure 1).

Of the three screening pathologies addressed in the study, cervical cancer stands out in Argentina, since around 4,000 new cases are diagnosed annually and 1,800 women die from the disease [19]. Furthermore, this type of cancer is a stark reflection of social inequality in health, as it continues to be one of the main causes of death in low-income countries, despite being potentially eradicable. Argentina has implemented a comprehensive strategy for uterine cervix cancer prevention [20] that includes primary prevention through the HPV vaccine, and secondary prevention, based on screening (PAP or HPV test).

The decrease in the number of PAPs in the province of Santa Fe and consequently in the coverage of this screening line, added to the low coverage in the pre-pandemic period, could have consequences in relation to later diagnoses of cervical cancer, with a decrease in the possibilities of a cure for these patients. If we exclusively analyse variable number two, i.e. the total number of PAPs with unsatisfactory results, which has an absolute variation of 265 fewer studies between both time frames, we may conclude that in the 2020 3-month period under study, 265 patients with some alteration in the cervix were not diagnosed (CIN2 or more).

Breast cancer, the most common malignancy, presents a similar pattern with the highest mortality in the province. The goal of screening programmes is to reduce mortality, and so far mammography has been the only screening method proven to be effective in meeting this goal.

It is estimated that the decrease in coverage levels will be associated with an increase in mortality [21], although more specific studies with a different methodology are required. In this study, the reduction in mammograms detected between both periods was associated with a high proportion of positivity in the second period. This is due to the official recommendations of the Ministry of Health of the Province and the National Cancer Institute, to prioritise for mammographic screening in high-risk patients. The selection of indications for complementary studies in this group of patients possibly determined that the percentage of positivity is higher in the intra-pandemic period.

Colorectal cancer screening with iFOBT is the most recent programme in the province. It presented an escalation during 2019, incorporating screening with iFOBT in several provincial primary care centres. The impact of the pandemic on these variables was significant, with a 90% reduction in the variables analysed, which also suggests later diagnoses of those cancers not diagnosed in that period.

Regarding the diagnostic section, although there was a reduction in the number of positive biopsies, in part due to the decrease in early detection, this reduction was lower than in the screening section. A reduction of 40.17% was observed in the total number of biopsies carried out in the province and a reduction in the number of biopsies with positive results, but the percentage of positivity was 22.72%. This can be explained by the selection of patients with high suspicion of cancer in the intra-pandemic period, which results in fewer studies but proportionally more positive results.

The deep commitment of the healthcare team is evidenced in the treatment segment. With a minimal difference in the pre-pandemic and intra-pandemic periods, the number of treatments carried out in both periods did not decrease in the provincial healthcare system, suggesting that access was guaranteed even during the lockdown, such as restricted public transport, saturation of services and attenuation of healthcare. This leads us to reflect on the patients who had already had contact with the healthcare system, and were able to continue their care. Meanwhile, we were unable to contact the subjects we had to monitor, invite and follow-up from the healthcare system, the presumably healthy individuals. In this study, no confounding factor was evaluated due to the clear association of the results found with the COVID-19 pandemic.

The limitations of this study were the lack of complete data in the registries, a problem identified throughout the country and Latin America. Neither were other variables from the cancer care line included that could improve the diagnosis of the situation, such as oncological surgeries performed or palliative treatment. However, no official records to carry out these evaluations are available.

In the current situation of the country with a sustained increase in the number of cases, waiting for a next growing wave, there are possible strategies to guarantee the continuity of the cancer control, especially in terms of screening.

Scientific evidence indicates that cervical cancer occurs more commonly in women from the age of 40, peaking around the age of 50. Therefore, according to the national guidelines, a fundamental concept is to determine the age of the target population in the context of limited resources. Screening should focus on women between 35 and 40 years of age, and the other age groups should be postponed. Therefore, a strategy to improve cervical cancer screening outcomes is to focus on screening this age group where the expected benefit is greater [22]. Population screening with the HPV test and self-collection modality might be a useful alternative in this epidemiological situation.

With regard to colon screening lines, home delivery of the collection and reading kit might be implemented, as well as teleconsultation to guide the reading and result process.

As for breast screening, scheduled shift systems might be implemented during hours of low patient circulation in the effectors, as well as separating the mammography equipment from the rooms and circulation of COVID-19 patients.

## Conclusion

In this study, we present reductions in cancer care at all levels, but predominantly in the screening and early diagnosis sections. In some situations, the percentages of positivity were higher in the intra-pandemic period, which accounts for a more specific selection of patients with high risk or high suspicion, for example, to perform mammograms or biopsies, thus avoiding the circulation of patients in the services

Patients already diagnosed and undergoing specific cancer treatment maintained their care even with the complications and barriers typical of the lockdown.

Addressing the health challenges posed by chronic pathologies and providing cancer patients with continuity of medical care and diagnosis, while responding to the global situation of the pandemic, will require strong, organised and resilient healthcare systems, as well as new innovative cancer management strategies.

## List of Abbreviations

ASCUS: Atypical Squamous Cells of Undetermined Significance
VA: Absolute variation
CCA: Cancer Control Agency
iFOBT: Immunochemical Faecal Occult Blood Test
NCCP: National Cancer Control Plan
PAP: Papanicolaou test, PAP smear
RECASFE: Santa Fe Cancer Registry
SITAM: National Screening Information System (Sistema de Información de Tamizaje)

## Authors’ contributions

All authors made substantial contributions to the conception and design of the study, acquisition or analysis of data, and revision of the report. All authors have been involved in drafting the manuscript or revising it critically for important intellectual content.

Final approval of manuscript: All authors

Accountable for all aspects of the work: All authors

## Conflicts of interest

The authors declare that they have no conflicts of interest.

## Funding statement

The group of researchers declares that they did not receive any type of sponsorship or funding for the preparation of this work.

## Figures and Tables

**Table 1.** Pooled results of the variables.

Section of the line of care	Variable	Period 1 (*n*)	Period 2 (*n*)	AV	RV (%)	CI (95%)	*p* value
Screening	PAP performed (*n*)	50,119	21,695	−28,424	(−) 56.71	56.28−57.15	
	PAP performed with unsatisfactory results (*n*)	285	20				
	% positivity	0.57	0.09	−0.48	(−) 83.79		<0.0001
	Number of mammograms performed (*n*)	9,918	2,098	−7,820	(−) 78.85	78.03–79.65	
	Number of mammograms performed with unsatisfactory results (*n*)	60	51				
	% positivity	0.60	2.43	1.83	301.83		<0.0001
	iFOBT performed (*n*)	418	51	−367	(−) 87.80	84.54−91.06	
	iFOBT performed with positive results (*n*)	132	12				
	% positivity	31.58	23.53	−8	−25.49		0.3097
Diagnostic	Biopsies performed (*n*)	12,515	7,488	−5,027	(−) 40.17	39.31–41.03	
	Biopsies with a positive result for cancer (*n*)	1,257	923				
	% positivity	10.04	12.33	2	22.72		<0.0001
Specific treatment	Number of oncological prescriptions (*n*)	3,036	2,940	−96	−3.16	2.52–3.80	

Own elaboration; databases exposed in methodology

AV = Absolute variation; VR = Variation rate; PAP = Papanicolau; iFOBT = Immunochemical faecal occult blood test
